# Analgesic Medication in Fibromyalgia Patients: A Cross-Sectional Study

**DOI:** 10.1155/2022/1217717

**Published:** 2022-09-22

**Authors:** H.-C. Aster, D. Evdokimov, A. Braun, N. Üçeyler, C. Sommer

**Affiliations:** ^1^Department of Neurology, University Hospital Würzburg, Würzburg 97080, Germany; ^2^Department of Child and Adolescent Psychiatry, Psychotherapy and Psychsomatics, University Hospital Würzburg, Würzburg 97080, Germany

## Abstract

There is no approved drug for fibromyalgia syndrome (FMS) in Europe. In the German S3 guideline, amitriptyline, duloxetine, and pregabalin are recommended for temporary use. The aim of this study was to cross-sectionally investigate the current practice of medication in FMS patients in Germany. We systematically interviewed 156 patients with FMS, while they were participating in a larger study. The patients had been stratified into subgroups with and without a decrease in intraepidermal nerve fiber density. The drugs most commonly used to treat FMS pain were nonsteroidal anti-inflammatory drugs (NSAIDs) (41.0% of all patients), metamizole (22.4%), and amitriptyline (12.8%). The most frequent analgesic treatment regimen was “on demand” (53.9%), during pain attacks, while 35.1% of the drugs were administered daily and the remaining in other regimens. Median pain relief as self-rated by the patients on a numerical rating scale (0–10) was 2 points for NSAIDS, 2 for metamizole, and 1 for amitriptyline. Drugs that were discontinued due to lack of efficacy rather than side effects were acetaminophen, flupirtine, and selective serotonin reuptake inhibitors. Reduction in pain severity was best achieved by NSAIDs and metamizole. Our hypothesis that a decrease in intraepidermal nerve fiber density might represent a neuropathic subtype of FMS, which would be associated with better effectiveness of drugs targeting neuropathic pain, could not be confirmed in this cohort. Many FMS patients take “on-demand” medication that is not in line with current guidelines. More randomized clinical trials are needed to assess drug effects in FMS subgroups.

## 1. Introduction

Fibromyalgia syndrome (FMS) is a chronic pain disorder associated with fatigue, sleep, memory, and mood disturbances, defined by a widespread pain index (WPI) and the symptom severity scale, symptom persistence over 3 months, and exclusion of all other diseases that might cause pain [1]. The etiology of FMS is still largely unknown. The majority of patients are women [2].

Systematic reviews of randomized clinical trials have shown that serotonin–norepinephrine reuptake inhibitors (SNRIs) [3], pregabalin [4], noradrenaline reuptake inhibitors (NRIs) [5], tricyclic antidepressants [6], and cyclobenzaprine [7] have a small but significant effect on FMS pain severity. Opioids or dopaminergic agents had no effect on pain and carry the risk of drug dependency [5]. In the German S3 guideline of 2017 [8] and the European Alliance of Associations for Rheumatology (EULAR) recommendations of 2016 [9], amitriptyline, duloxetine, and pregabalin are recommended as temporary drug therapies for FMS. The Canadian and Israeli guidelines advise to use SNRIs and anticonvulsants (pregabalin and gabapentin) [10, 11]. All guidelines also point out that nonpharmacological therapy such as aerobic training or cognitive-based behavioral therapy may be more efficient in the relief of pain and fatigue, with fewer side effects.

No drug is licensed specifically for FMS in Europe, while the United States Food and Drug Administration approved pregabalin, duloxetine, and milnacipran for this indication [12]. In Europe, the European Medical Agency (EMA) has approved amitriptyline for the treatment of neuropathic pain as part of multimodal treatment, tramadol for moderate-to-severe musculoskeletal pain [13], strong opioids for cancer pain and chronic noncancer pain as a last therapeutic option, and pregabalin and gabapentin for the treatment of neuropathic pain.

We have prospectively recruited and comprehensively investigated a large cohort of patients with FMS [14]. Here, we were interested in how these patients were medically treated in the absence of specifically licensed drugs and in the context of current guidelines. We report the current pharmacological treatment of these patients, which drugs were discontinued and why, and how well the individual drugs reduced pain. Previously, we showed that FMS patients with small fiber pathology as indicated by reduced intraepidermal nerve fiber density (IENFD) at the lower and upper legs had more severe clinical symptoms [14]. Hence, we hypothesized that drug efficiency might differ in patient subgroups stratified for small fiber pathology reflecting a potential neuropathic component.

## 2. Materials and Methods

**Figure 1:**
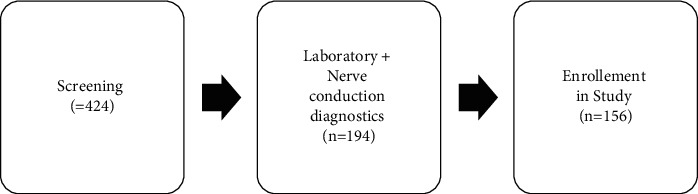
Flowchart of the inclusion process of patients.

Patients were recruited for a larger study on FMS and small fiber pathology at the Department of Neurology, University Hospital Würzburg, Germany, between 2014 and 2019. A flowchart of the inclusion process is shown in Figure 1. The study was approved by the Ethics Committee of the University of Würzburg Medical Faculty (number 121/14), and all study participants gave written informed consent. Before study inclusion, all patients were diagnosed by a board-certified rheumatologist. All patients were then examined by a neurologist, and a structured medical history focusing on pain and current and former FMS treatment was recorded. All patients were diagnosed according to the 1990 and 2010 criteria of the American College of Rheumatology [15] after alternative diagnoses had been excluded [14]. The exclusion criteria included amongst others a diagnosis of a manifest psychiatric or neurological disease, possible somatic underlying causes of neuropathy or other pain disorders, and a history of cancer in the last 5 years. Further details on the recruitment and exclusion criteria can be found in [14, 16]. Patients were asked about their current medication, the indication, the dose, the effect, and treatment regimen. Furthermore, the medication history was taken, and the reasons why previous medication was discontinued were elicited. These data were stored electronically in standardized forms. Since many patients took several pain medications, data are given relative to the total number of patients' replies to a specific drug. Only the general frequency of medication classes used in Table 1 is given relative to the absolute number of patients (Table 1).

Having determined IENFD in skin biopsies of the lower and upper leg, we had identified patients at the two opposite ends of the spectrum, which resulted in a group with pathologic IENFD in both the distal and the proximal biopsy and a group with normal IENFD in both biopsies [14]. Here, we investigated whether drug intake and efficacy differed between these previously determined subgroups.

To evaluate pain relief by the drugs, we used a numeric rating scale (NRS, 0–10; 0 = no pain; 10 = worst possible pain). This scale was used for all analyses regarding the effectiveness of individual drugs in relieving pain. The remaining pain questionnaires were only used to obtain a more comprehensive clinical characteristic but were not related to the effectiveness of the medications. To evaluate persistent pain severity, we used the Graded Chronic Pain Scale (GCPS), which reflects two dimensions of chronic pain: pain intensity and pain-related disability [17]. To assess the presence of depressive symptoms, we used the “Allgemeine Depressionsskala” (ADS), which is a German version of the Center for Epidemiological Studies-Depression Scale questionnaire [18]. To evaluate the extent of catastrophizing, we applied the Pain Catastrophizing Scale [19], We further used the State-Trait Anxiety Inventory (STAI) [20], which is a commonly used measure of trait and state anxiety. In order to record the impact of FMS symptoms on everyday life activities, we used the Fibromyalgia Impact Questionnaire (FIQ) [21]. The O'Leary-Sant symptom and problem index assesses the impairment by bladder dysfunction [22]. Since some patients also report problems or pain during urination, we used this questionnaire to evaluate secondary symptoms and possible side effects.

We categorized diclofenac, ibuprofen, and acetylsalicylic acid as nonsteroidal anti-inflammatory drugs (NSAIDs); etoricoxib and nimesulide as cyclooxygenase-2 (COX-2) inhibitors; tilidine and tramadol as weak opioids; oxycodone, tapentadol, and fentanyl as strong opioids; tolperisone as a muscle relaxant; fluoxetine and sertraline as selective serotonin reuptake inhibitors (SSRI); and duloxetine as serotonin–norepinephrine reuptake inhibitors (SNRI). Some patients reported guaifenesin treatment explicitly against FMS symptoms; hence, we also included this mucus diluent in our analysis.

For statistical analysis, the program IBM SPSS Statistics for Windows version 25.0 (IBM Corp. Armonk, NY, USA) was used. Data were converted into the dichotomic multiple answer system of SPSS and evaluated using crosstabs. the Shapiro–Wilk test was performed to check for normal distribution of the data. For normally distributed data (all questionnaires except the GCPS and the STAI), we used a two-sided *t*-test for group comparisons. For nonnormally distributed data, the group comparison was performed by the Mann–Whitney *U* test. The crosstabs were tested for significance using the chi-square test. Correlation analysis was performed by the two-sided Spearman–Rho test. The confidence interval was 0.95, and the significance threshold was *p* < 0.05. In order to compare the effectiveness of the pain medication between the small nerve fiber groups, only medication classes that were taken by more than 15 patients were included for sufficient statistical power.

## 3. Results

One hundred and fifty-six patients (144 women, 12 men) were included in our analysis. The median age was 50.6 years (range 21.5–74.7). The sum scores of the patients' symptom questionnaires and the proportion of frequent FMS comorbidities are displayed in the Supplementary Table 1. There was no difference between the groups with and without pathologic IENFD in the results of the questionnaires (Supplementary Table 2).

### 3.1. Current Medication

The most frequently taken class of drugs was NSAIDs with 41.0% of all patients, followed by metamizole with 22.4% and amitriptyline with 12.8% (Table 1). Opioids were taken by 7.7% of the patients. 16% of the patients in our study did not take any medication against FMS symptoms. The most frequent analgesic treatment regimen was “on demand” during pain exacerbations (53.9% of all prescribed drugs), while 35.1% of the drugs were administered according to a fixed regime. Antidepressants were mostly taken on a daily basis (Table 2). 57.6% of patients took one analgesic drug, 27.3% two drugs, 5.2% three drugs, and 1.2% four drugs.

Only 29.6% of the patients had drug therapy according to the German S3 guideline. However, 78.8% of the patients had already tried at least one of the drugs recommended in the guideline in the past and had discontinued it due to side effects or lack of efficacy. Amitriptyline (37.7% of all patients) was the most frequently discontinued drug in the past, followed by NSAIDs (35.1%) and pregabalin (19.2%). Current therapy was the first medical treatment attempt for only 9.1% of the patients. Most often, previous drugs had been discontinued due to lack of effect (63.7% of all prescribed drugs). The median duration of the current drug therapy up to study enrollment was 3 years (range from 1 month–30 years).

### 3.2. Pain Relief by Type of Medication

The patients were asked to rate the pain reduction by the individual drugs on an NRS of 0–10. We analyzed all drug classes taken by *n* > 15 patients. These were NSAIDs with a median pain reduction of 2 points (range 0–5), SNRIs with a median of 1 point (range 0–3), amitriptyline with 1 point (range 0–4), and metamizole with 2 points (range 0–8) (Table 3).

### 3.3. Pain Relief in Patient Subgroups

We compared the groups with prominent small fiber pathology (reduction of IENFD in distal and proximal biopsy, *n* = 36) and with entirely normal skin innervation (*n* = 42). In the overall response and also analyzing the frequently taken drugs NSAIDs or metamizole, we did not find intergroup differences in treatment response (Table 4).

### 3.4. Reasons for Discontinuing Previous Medication

33.7% of patients had already used other drugs before their current therapy, 25.5% two drugs, and 29.1% three drugs. Lack of efficacy was the most frequently mentioned reason for discontinuing past treatment with opioids, NSAIDs, SSRIs, flupirtine, and acetaminophen. Intolerable side effects were the most frequently mentioned reason to discontinue SNRI, amitriptyline, and pregabalin (Table 5).

### 3.5. Correlations between Medication and Clinical Symptoms

We hypothesized that the choice of drug might be guided by symptom, severity, and phenotype. For example, patients with more severe pain might more often be prescribed opioids, and patients with a more “neuropathic” phenotype might more often receive antineuropathic drugs. This was not the case.

We found several correlations between the intake of distinct drugs and clinical parameters (Table 6). Intake of SNRIs (*r* = -0.25) or guaifenesin (*r* = -2.0) was negatively correlated with the IENFD in the distal leg. Interestingly, the use of strong opioids was associated with higher scores in the “O ´Leary/Sant voiding and pain indices.” To validate this correlation, we conducted a direct group comparison. In this direct comparison of the questionnaire results between patients taking opioids (*n* = 12) and those not taking any (*n* = 146), we found one difference, namely, higher scores (*p*=0.02) in the “O ´Leary/Sant voiding and pain indices,” which asks about urinary problems. Since these correlation analyses had an exploratory purpose to enable us to test hypotheses from them later in large cohort studies, we did not apply the Bonferroni correction. These data should therefore be regarded as pilot results and warrant replication.

### 3.6. Dosage of FMS Analgesic Medication

Only 29.6% of FMS patients took recommended medication according to the German FMS guideline [8]. In the group of patients taking pregabalin, 25% used the recommended dosage of 150–450 mg/day, while 75% of patients used a lower dose (median 75 mg/d, range 25–500 mg/d). For amitriptyline, recommended doses between 10 mg/d and 50 mg/d were used by 84.2% patients, in 10.5% of cases, the dose was lower, and in 5.3% of cases, the dose was higher (median 25 mg/d, range 10–75 mg/d). Two patients took an SSRI such as fluoxetine (recommended dosage 20–40 mg/d) for an accompanying depressive disorder: one of these patients was underdosed (10 mg/d) and the other overdosed (50 mg/d). We did not detect any overdoses in our cohort for the frequently used drugs: metamizole (maximum recommended dose 4000 mg/d), COX-2 inhibitors such as etoricoxib (maximum recommended dose 120 mg/d) and acetaminophen (maximum recommended dose 4000 mg/d), and NSAIDs, such as ibuprofen (maximum recommended dose 2400 mg/d).

### 3.7. Medication due to Comorbidities

As shown in Table 7, 22.9% of the patients had no other comorbidities requiring drug treatment. The three most frequently treated comorbidities were thyroid dysfunction (16.7%), arterial hypertension (13.2%), and depression (7.6%). Table 7 shows the respective medication that was taken for each of these conditions. The most commonly taken drugs were l-thyroxine (14.8%), proton pump inhibitors (5.5%), and vitamin *D* (5.9%); drugs are listed in Table 7. Some drugs such as SSRIs that might also be used for the treatment of FMS symptoms, in these cases, were explicitly prescribed for other indications, e.g., depression.

## 4. Discussion

In this cross-sectional study of 156 patients with FMS, we found that NSAIDs and metamizole on demand were the most frequently used drugs. Drugs with proven efficacy in randomized controlled trials (RCTs) and with recommendations in national and international guidelines [8] were only used by 29.6% of the patients (amitriptyline 12.8%, pregabalin 5.1%, and duloxetine (SNRI) 11.5%). Other drugs with efficacy in RCTs such as milnacipran were not encountered in our cohort. Over the course of their disease, more patients had been using either amitriptyline (37.7%) or pregabalin (19.2%); however, these drugs had been discontinued due to lack of efficacy or side effects.

Among the few studies worldwide that have investigated the current use of drugs in FMS, one explicitly deals with opioids. A study from the United States of America (USA) examined the intake of opioids by FMS patients from 2011 to 2017 [23]. In 2011, 42% of FMS patients were taking opioids as pain medication, but in 2016, the rate had dropped to 27%, probably due to higher awareness towards the side effects and addictive potential of opioids. The second study was also based on the USA and investigated multimorbidity and polypharmacy in elderly FMS patients [24]. The authors described that the most frequently taken drugs were sleeping aids with 33.3%, SSRIs with 28.7%, and SNRIs with 21.0%. In this study, opioids accounted for 22.4% of all drugs.

Two population-based studies focused on the choice of drug against FMS symptoms [25, 26]. Both studies examined cohorts in the USA, one of which showed that less than 20% of the drug therapies were retained for more than a year [26]. More than 50% of the patients in this study took opioids. At treatment initiation, the average daily dose of pregabalin was 75 mg/d, and in 52% of patients treated with pregabalin, this dose was not increased. Of these 52%, 78% discontinued pregabalin within 3 months. This shows some similarities with our data, since we also see a relatively low dosing of pregabalin. One explanation for the retention of pregabalin at higher doses may be that higher doses are more effective and thus lead to a longer duration of treatment. The second study examined the factors influencing the prescription of drugs in FMS patients with a focus on duloxetine and found that, among other factors, prior intake of pregabalin made the prescription of duloxetine more likely [25].

Our cohort is smaller compared to the previously mentioned studies, but similar in patients' characteristics. Here, as well, the average age is approximately 50 years, and on average, about 80% of the patients are women. However, the number of other pain disorders was lower in our cohort compared to others [25]. This may be due to our relatively strict exclusion criteria [14]. Our patients were extensively examined rheumatologically and neurologically for other possible causes of pain until the diagnosis of FMS was made. Furthermore, the proportion of opioids was 7.7%, which is lower than in the US-American cohorts with up to 50%. The reason may be a higher sensitivity to opioid related problems, stricter prescription rules [27, 28], and adherence to guidelines [29]. In contrast to the abovementioned studies, however, our patients were all volunteers in a prospective study, so our patient population may be less severely affected than those studied in pain clinics or population studies, more aware of nondrug therapies, and motivated for treatment.

The reason for the low number of patients taking the drugs according to the guidelines (29.6%) remains unclear. One obvious reason may be that duloxetine and pregabalin are off-label for FMS in Germany. Other reasons may be lack of information in the group of the treating physicians or that physicians decided to discontinue an ineffective drug therapy after consulting the guidelines, which recommend initial nondrug therapy. Another reason may be a lack of adherence by patients. Often the term “antidepressants” is misunderstood and patients feel stigmatized by taking such a drug. Many patients also report side effects, such as weight gain or fatigue, which can lead to severe loss of quality of life. We show in our results that this varies greatly depending on the medication taken. This may lead to patients preferring complementary medicine to classical medicine. Since there are no drugs specifically approved for FMS in Europe, a standardized therapy is more difficult. Well-planned RCTs or register studies might lead to additional safety and possibly to the licensing of helpful drugs in Europe.

Although the German guidelines explicitly do not recommend the use of opioids in FMS [8], 7.7% of the patients were taking them. Our correlation analysis found an increased number of problems during urination in these patients. This might be explained by an opioid side effect on the detrusor muscle [30, 31]. Our hypothesis that there are differences in the intake and efficacy of the drugs between the subgroups with “neuropathic” and “nonneuropathic” pain, as evaluated by the presence or absence of small fiber pathology, could not be confirmed; however, our subgroups were too small to exclude such an effect. The question should be investigated in a prospective study with a larger number of cases with the goal to provide more personalized therapy.

Our study has a number of limitations. For certain classes of drugs, the number of patients was low, so the conclusions in these cases are limited. This is a cross-sectional study; therefore, the recall of medication effects may be biased. We did not query the compliance of the patients, which could have been influential on the results. Patients were asked to distinguish between multiple medications individually; however, overlapping effects may have occurred. In addition, the different drugs were taken over different periods of time; we could not control this parameter with our data. Previously published small RCTs do not show a superiority of NSAIDs over the placebo effect [32]; however, the fixed regime in the RCTs cannot be compared with the on-demand application by our patients, and the impact of a placebo effect in our cohort is unclear. Furthermore, the question whether the correlations between the intake of certain drugs and patient reported symptoms reflect medication side effects or insufficiently treated FMS symptoms cannot be answered by our cross-sectional study.

## 5. Conclusions

In conclusion, FMS patients in Germany take many different medications for their pain, which are not officially recommended for the treatment of FMS. However, these lead to moderate therapeutic success. These substances, such as NSAIDs and metamizole, should be tested in randomized controlled clinical studies in FMS. To assess possible differences in therapeutic response between the subgroups with and without small nerve fiber pathology, studies with larger cohorts are needed. Physicians treating FMS patients should also pay attention to the recommended dose ranges with regard to the tolerability of the medication. Limitations of the study were the small number of patients in the subgroups, the cross-sectional design that did not allow for conclusions about placebo effects or overlapping effects with multiple medications, and a lack of control for patients' medication adherence.

## Figures and Tables

**Table 1:** Current medication of fibromyalgia patients (total number of patients = 156) and previously discontinued medication. Some of the patients took more than one medication.

Medication	Current use Number of patients currently using the drug (% of all patients)	Past use Number of patients having used the drug in the past (% of all patients)
NSAID	64 (41.0)	53 (35.1)
Metamizole	35 (22.4)	14 (9.3)
None	25 (16.0)	26 (17.2)
Amitriptyline	20 (12.8)	57 (37.7)
SNRI	18 (11.5)	33 (21.9)
Weak opioid	9 (5.8)	27 (17.9)
COX-2 inhibitor	8 (5.1)	2 (1.3)
Pregabalin	8 (5.1)	28 (19.2)
Muscle relaxant	7 (4.5)	3 (2.0)
Acetaminophen	6 (3.8)	12 (7.9)
Cannabinoid	4 (2.6)	—
Strong opioid	3 (1.9)	5 (3.3)
Guaifenesin	3 (1.9)	—
Triptan	3 (1.9)	—
Flupirtine	3 (1.9)	13 (8.6)
SSRI	3 (1.3)	11 (7.3)
Corticosteroid	1 (0.6)	1 (0.7)
Lidocaine	1 (0.6)	1 (0.7)
Magnesium	1 (0.6)	—
Mirtazapine	1 (0.6)	—

**Table 2:** Treatment regimens for each category of medication.

	On demand (%)	Fixed daily regime (%)
NSAID	97.0	3.0
Metamizole	82.4	17.6
Amitriptyline	5.3	94.7
SNRI	0.0	100.0
Weak opioid	55.6	44.4
Pregabalin	0.0	100.0
Strong opioid	14.0	86.0
COX-2 inhibitor	71.4	28.6
Muscle relaxant	28.6	71.4
Acetaminophen	100.0	0.0
Cannabinoid	25.0	75.0
Guaifenesin	0.0	100.0
Triptans	100.0	0.0
Flupirtine	100.0	0.0
SSRI	0.0	100.0
Corticosteroid	100.0	0.0
Lidocaine	0.0	100.0
Magnesium	0.0	100.0
All	60.3	39.4

**Table 3:** Effect of the medication on pain relief.

	Percentage of patient replies indicating pain reduction by *x* points on the NRS with a given drug (retrospective evaluation).									*N*
	0	1	2	3	4	5	6	8	Pain reduction in NRS (median, range)	
NSAID	6.2	18.5	38.5	16.9	16.9	3.1	0.0	0.0	2.3 (2, 0–5)	64
Metamizole	12.1	15.2	51.5	15.2	0.0	3.0	0.0	3.0	2.0 (2, 0–8)	33
Amitriptyline	45.0	15.0	15.0	20.0	5.0	0.0	0.0	0.0	1.3 (1, 0–4)	20
SNRI	38.9	33.3	16.7	11.1	0.0	0.0	0.0	0.0	1.0 (1, 0–3)	18
Drugs taken by < 15 patients										
Weak opioid	0.0	22.2	44.4	0.0	22.4	11.0	0.0	0.0	2.6 (2, 1–5)	9
Pregabalin	12.5	0.0	50.0	37.5	0.0	0.0	0.0	0.0	2.1 (2, 0–3)	8
Strong opioid	0.0	14.3	0.0	71.4	0.0	14.3	0.0	0.0	3.0 (3, 1–5)	7
COX-2 inhibitor	0.0	14.3	28.6	42.9	14.3	0.0	0.0	0.0	2.6 (3, 1–4)	7
Muscle relaxant	33.3	16.7	33.3	0.0	16.7	0.0	0.0	0.0	1.5 (2, 0–4)	6
Acetaminophen	16.7	16.7	33.3	16.7	16.7	0.0	0.0	0.0	2.0 (2, 0–4)	6
Cannabinoid	0.0	0.0	25.0	25.0	25.0	0.0	25.0	0.0	3.7 (4, 2–6)	4
Guaifenesin	33.3	0.0	0.0	0.0	33.3	0.0	33.3	0.0	3.3 (4, 0–6)	3
Flupirtine	0.0	33.3	33.3	0.0	33.3	0.0	0.0	0.0	2.3 (2, 1–4)	3
SSRI	0.0	50.0	0.0	50.0	0.0	0.0	0.0	0.0	2.0 (2, 1–3)	2
Triptans^1^	0.0	0.0	0.0	50.0	50.0	0.0	0.0	0.0	3.5 (3.5, 3-4)	2
Corticosteroid	0.0	0.0	0.0	0.0	100.0	0.0	0.0	0.0	4.0 (4)	1
Lidocaine	0.0	0.0	0.0	0.0	100.0	0.0	0.0	0.0	4.0 (4)	1
All									2.1 (2, 0–8)	195

*N*, the number of patients' replies when asked about a given drug. ^1^Used in migraine attacks.

**Table 4:** Effect of the medication categories (current treatment) on pain relief in NRS-points, in the subgroups with and without reduction of skin innervation.

	Reduced IENFD		Normal IENFD		*P*	All	
	*N*	Response (median, range)	*N*	Response (median, range)		*N*	Response (median, range)
NSAID	16	2, 0–5	21	2, 0–4	0.33	65	2, 0–5
Metamizole	9	2, 0–3	5	2, 1–3	0.36	33	2, 0–8
Amitriptyline	5	0, 0–2	6	1, 0–4	0.24	20	1, 0–4
SNRI	8	1, 0–2	3	0, 0	0.13	18	1, 0–3

*N*, the number of patients replies when asked about a given drug; IENFD, normal and reduced intraepidermal nerve density.

**Table 5:** Reasons for discontinuing medication given in % of treatment episodes.

	No effect (%)	Side effects (%)	No reason given (%)	*N*
Amitriptyline	42.3	57.7	8.8	57
NSAIDs	83.7	16.3	7.5	53
SNRI	42.4	57.6	0	33
Pregabalin	48.3	51.7	0	29
Weak opioids	74.1	25.9	0	27
Metamizole	57.1	28.6	14.3	14
Flupirtine	84.6	7.7	7.7	13
Acetaminophen	100.0	0.0	0	12
SSRI	81.8	18.2	0	11
Strong opioids	60.0	40.0	0	5
COX-2 inhibitors	100.0	0.0	0	2
Cyclobenzaprine	33.3	33.3	33.3	3
Corticosteroids	100.0	0.0	0	1
Lidocaine	100.0	0.0	0	1
All	60.1	34.1	5.8	261

*N*, the total number of treatments with the respective drug in the past.

**Table 6:** Correlations between the use of certain classes of medication and clinical symptoms and the IENFD in the lower leg.

Medication	Questionnaire (CC; *p* value)			

No medication	STAI (0.18; 0.02)			

Weak opioid	GCPS disability due to pain (−0.16; 0.03)			

Strong opioid	O' Leary (0.23; 0.005)			

NSAID	NPSI (0.17; 0.02)	GCPS grade (0.19; 0.01)	ADS (0.2; 0.01)	

SNRI	Pain Catastrophizing Scale (0.18; 0.02)	FIQ (0.016; 0.04)	O′ Leary (−0.2; 0.01)	IENFD lower leg (−0.25; 0.001)

Muscle relaxant	GCPS disability due to pain (−0.15; 0.04)	ADS (−0.1; 0.04)		

Guaifenesin	IENFD lower leg (−0.2; 0.01)			

Flupirtine	Paresthesia (0.2; 0.01)			

**Table 7:** Concomitant medications and their indications.

Indication	Generic	*N*	%
None	None	52	22.9

Thyroid dysfunction	L-Thyroxin	35	14.9
	Iodine	3	1.4

Hypertension	Beta-blocker	12	5.3
	Angiotensin-converting enzyme (ACE) inhibitor	6	2.6
	Angiotensin II blocker	4	1.8
	Calcium channel blocker	5	2.2

Depressive symptoms	SSRI	11	4.8
	SNRI	3	1.4
	Herbal agent	2	0.8

Sleep disturbances	Tricyclic antidepressant	7	3.0
	Zopiclone	1	0.5
	SSRI	1	0.5
	Pregabalin	1	0.5

Stomach pain	Proton pump inhibitor	16	7.0

Vitamin substitution	Vitamin D	13	5.7
	Estrogen	3	1.4

Asthma	Beta II agonist	11	4.6
	Corticosteroid	7	3.1

Other pain disorder	NSAID	2	0.9
Anxiety symptoms	SSRI	1	0.5
Osteoporosis	Vitamin D	1	0.5

Others		32	14.1

*N*, the number of treatment regimens; %, percentage of the whole cohort.

## Data Availability

The data used to support the findings of this study are available from the corresponding author upon request.

## Supplementary Materials

Supplementary Materials Supplementary Table 1: clinical characteristics of the cohort and proportion of FMS comorbidities. Supplementary Table 2: clinical and questionnaire data compared between subgroups according to IENFD.
