# Supplementary material for: Analgesic Medication in Fibromyalgia Patients: A Cross-Sectional Study
Source: Pain Res Manag. 2022 Sep 22;2022:1217717. doi: 10.1155/2022/1217717 (PMC9553668; doi:10.1155/2022/1217717)
Supplement: Supplementary Materials — Supplementary Table 1: clinical characteristics of the cohort and proportion of FMS comorbidities. Supplementary Table 2: clinical and questionnaire data compared between subgroups according to IENFD. [file 1217717.f1.docx]

| **Clinical characteristics (n=156)** | **Median (Range)** |
| --- | --- |
| **Age** | 50.6 ( 21.5- 74.7) |
| **Time since diagnosis (years)** | 4 (0-35) |
| **Duration of pain due to the disease (years)** | 13 (0.8-56) |
| **Current pain intensity (NRS 0-10)** | 5 (1-9) |
| **NPSI sum score** | 0.41 (0.11-0.91) |
| **GCPS Pain intensity** | 70 (0-90) |
| **GCPS Disability due to pain** | 60 (10-266) |
| **Pain Catastrophizing sum score** | 23 (3-49) |
| **ADS Score** | 22 (3-51) |
| **FIQ Sum Score** | 49.3 (8.6-70.6) |
| **O’Leary-Sant Symptom and Problem Indexes (Painful bladder)** | 10 (0-33) |
| **STAI Sum score** | 43 (0-79) |
| **Overall response to analgesic medication (improvement in NRS 0-10)** | 2 (0-6) |
|  | |
| **Comorbidites (n=156)** | **Percentage (Absolute number)** |
| **Autoimmune disease** | 11.5 (18) |
| **Allergic disease** | 35.3 (55) |
| **Depressive symptoms** | 35,9 (56) |
| **Anxiety symptoms** | 10.3 (16) |

Supplementary Table 1: Clinical characteristics of the cohort and proportion of FMS comorbidities (STAI: State-Trait Anxiety Inventory, NPSI: Neuropathic Pain Symptom Inventory, GCPS: Graded Chronic Pain Scale, ADS: Allgemeine Depressionskala, FIQ: Fibromyalgia Impact Questionnaire)

|  | **PNS (n=36)** | **noPNS (n=50)** | **p-value** |
| --- | --- | --- | --- |
|  | **Mean±SD / Median (Range)** | **Mean±SD / Median (Range)** |  |
| **Age** | 51.3 (31.1-72.2) | 51.8 (21.6-69.2) | 0.87 |
| **IENFD lower leg (fibres/mm)** | 4.2 (0.9-5.3) | 8.3 (5.6-14.4) | **< 0.001** |
| **IENFD upper thigh (fibres/mm)** | 5.4 (1.9-8.4) | 10.7 (8.6-20.0) | **< 0.001** |
| **NPSI sum score** | 43±16 | 39±15 | 0.24 |
| **GCPS pain intensity** | 67.7±15.1 | 63.3±13.5 | 0.63 |
| **GCPS disability due to pain** | 65 (10-83) | 51 (16.6-86.6) | 0.05 |
| **Pain Catastrophizing Scale** | 24.9±10.1 | 20.5±10.0 | 0.85 |
| **ADS** | 24.1±12.3 | 21.2±10.6 | 0.13 |
| **FIQ** | 46.9±12.4 | 44.0±11.9 | 0.39 |
| **The O’Leary-Sant Symptom Index and Problem Index** | 9 (0-33) | 8.5 (0-27) | 0.21 |
| **STAI** | 48.5 (0-68) | 41.5 (0-67) | 0.13 |

Supplementary Table 2: Clinical and questionnaire data compared between subgroups according to IENFD (intraepidermal nerve fiber density) (PNS: Reduced IENFD in biopsies from lower and upper leg, noPNS: Normal IENFD in biopsies from lower and upper leg.)

STAI: State-Trait Anxiety Inventory, IENFD: Intraepidermal nerve fibre density, NPSI: Neuropathic Pain Symptom Inventory, GCPS: Graded Chronic Pain Scale, ADS: Allgemeine Depressionskala, FIQ: Fibromyalgia Impact Questionnaire

When data were not normally distributed, median and the range are shown, and a Mann-Whitney U test was applied.
